# Phylogenomic analyses in Phrymaceae reveal extensive gene tree discordance in relationships among major clades

**DOI:** 10.1002/ajb2.1860

**Published:** 2022-06-05

**Authors:** Diego F. Morales‐Briones, Nan Lin, Eileen Y. Huang, Dena L. Grossenbacher, James M. Sobel, Caroline D. Gilmore, David C. Tank, Ya Yang

**Affiliations:** ^1^ Department of Plant and Microbial Biology University of Minnesota‐Twin Cities 1445 Gortner Avenue St. Paul Minnesota 55108‐1095 USA; ^2^ Systematics, Biodiversity and Evolution of Plants, Department of Biology I, Ludwig‐Maximilians‐Universität München Menzinger Strasse 67 80638 Munich Germany; ^3^ College of Life Science Henan Agricultural University 63 Nongye Road Zhengzhou Henan 450002 China; ^4^ Biological Sciences Department California Polytechnic State University, 1 Grand Avenue, San Luis Obispo California 93407 USA; ^5^ Department of Biological Sciences Binghamton University (State University of New York), 4400 Vestal Parkway E, Binghamton New York 13902 USA; ^6^ Department of Botany & Rocky Mountain Herbarium University of Wyoming, 1000 E. University Avenue, Laramie Wyoming 82071 USA

**Keywords:** *Diplacus*, *Erythranthe*, Lamiales, *Mimulus*, reticulate evolution, transcriptome, whole genome duplication

## Abstract

**Premise:**

Phylogenomic datasets using genomes and transcriptomes provide rich opportunities beyond resolving bifurcating phylogenetic relationships. Monkeyflower (Phrymaceae) is a model system for evolutionary ecology. However, it lacks a well‐supported phylogeny as a basis for a stable taxonomy and for macroevolutionary comparisons.

**Methods:**

We sampled 24 genomes and transcriptomes in Phrymaceae and closely related families, including eight newly sequenced transcriptomes. We reconstructed the phylogeny using IQ‐TREE and ASTRAL, evaluated gene tree discordance using PhyParts, Quartet Sampling, and a cloudogram, and carried out reticulation analyses using PhyloNet and HyDe. We searched for whole genome duplication (WGD) events using chromosome numbers, synonymous distances, and gene duplication events as evidence.

**Results:**

Most gene trees support the monophyly of Phrymaceae and each of its tribes. Most gene trees also support tribe Mimuleae being sister to Phrymeae + Diplaceae + Leucocarpeae, with extensive gene tree discordance among the latter three. Despite the discordance, the monophyly of *Mimulus* s.l. is rejected, and no individual reticulation event among the Phrymaceae tribes is well‐supported. Reticulation likely occurred among *Erythranthe bicolor* and closely related species. No ancient WGD was detected in Phrymaceae. Instead, small‐scale duplications are among potential drivers of macroevolutionary diversification of Phrymaceae.

**Conclusions:**

We show that analysis of reticulate evolution is sensitive to taxon sampling and methods used. We also demonstrate that phylogenomic datasets using genomes and transcriptomes present rich opportunities to investigate gene family evolution and genome duplication events involved in lineage diversification and adaptation.

With thousands of genes, phylogenomic datasets using genomes and transcriptomes are rich in information for not only clarifying phylogenetic relationships, but also identifying reticulate evolution, gene and genome duplications, and molecular evolution that contribute to macroevolutionary adaptation. However, detecting these events is difficult due to computational limitations, and studies often do not fully interrogate the data.

Monkeyflowers, as part of Phrymaceae, are a model system for evolutionary ecology (Wu et al., 2008; Twyford et al., 2015). With around 200 species, a primarily North American distribution, a rich history of ecological studies, and accumulating genomic resources, monkeyflower research has provided insights in speciation (Schemske and Bradshaw, 1999; Sobel, 2014), local adaptation (MacNair, 1983; Hall et al., 2010), pigment evolution (Streisfeld et al., 2013; Yuan et al., 2016; Ding et al., 2020), and development (Yuan, 2019). However, previous studies often focused on a single species or clades of closely related species (Stankowski and Streisfeld, 2015; Chase et al., 2017; Stankowski et al., 2019; Nelson et al., 2021), and we still lack a robust phylogenetic framework for the family. In addition, with phylogenetic uncertainty across Phrymaceae from previous analyses, the circumscription of *Mimulus* L. is under debate (Lowry et al., 2019; Nesom et al., 2019). Previous phylogenetic studies have established the polyphyly of *Mimulus* in its broad sense (Barker et al., 2012), and therefore we follow the narrow definition of *Mimulus* sensu Barker et al. (2012) that includes only seven species as part of the tribe Mimuleae, with the remaining species distributed into the genera *Diplacus* (tribe Diplaceae) and *Erythranthe* (tribe Leucocarpeae).

Previous molecular phylogenetic studies using Sanger sequencing have consistently supported five well‐supported tribes within Phrymaceae [Figure 1; the tropical Asian tribe Cyrtandromoeeae was only included in the sampling by Liu et al. (2020)]. However, phylogenetic relationships in Phrymaceae have been problematic due to discordance among analyses (Figure 1) that used: (1) different taxon sampling; (2) nuclear vs. plastome (cpDNA) markers, or even among cpDNA regions [*trnL‐F* only (Beardsley and Olmstead, 2002) vs. six cpDNA regions (Liu et al., 2020)]; and (3) different analytical approaches (maximum likelihood vs. Bayesian, ITS + ETS; Liu et al., 2020). In addition, despite previous and ongoing whole genome and exome sequencing efforts (Hellsten et al., 2013; Edger et al., 2017; Nelson et al., 2021), Phrymaceae still lacks a multi‐locus phylogenetic analysis using nuclear genes across major clades of the family.

**Figure 1:**
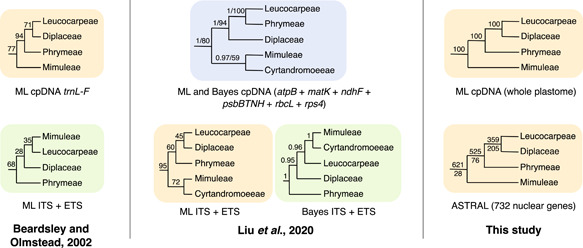
Summary of relationships among Phrymaceae tribes (Stevens, 2001 onwards) recovered by studies focusing on the backbone of the family. Numbers above branches are maximum likelihood (ML) bootstrap support (BS), Bayesian posterior probabilities (PP), or BS/PP. For the ASTRAL analysis, numbers above and below branches are the number of genes with concordant vs. discordant topologies that had MLBS > 50 in each gene tree. Trees shaded by the same color share compatible backbone topologies.

Macroevolutionary analyses using a large number of nuclear genes are powerful not only for inferring the phylogenetic relationships and history of reticulate evolution, but also investigating gene and genome evolution associated with lineage diversification and adaptation. Previous investigation of patterns of chromosome number changes across the North American members of Phrymaceae suggested extensive polyploidy events across the family (Beardsley et al., 2004). However, comparison of linkage maps established that the higher chromosome base number in *Erythranthe guttata* (Fisch. ex DC.) G.L. Nesom compared to *E. lewisii* (Pursh) G.L. Nesom & N.S. Fraga is due to chromosome fission and fusion instead of whole genome duplication (WGD; Fishman et al., 2014). The extent of WGD events (if any), and their location along the backbone of the family is still unexplored. Sampling of genomes and transcriptomes across major clades of Phrymaceae and analyzing both nuclear and cpDNA regions are needed to investigate the backbone structure in Phrymaceae and the genomic basis of the macroevolutionary diversification of Phrymaceae.

In this study we sampled transcriptomes and genomes covering four of the five tribes in Phrymaceae, including eight newly generated transcriptomes, to: (1) provide a phylogenetic backbone and examine gene tree discordance; and (2) investigate patterns of gene and genome duplication in the family. We found that most gene trees support Mimuleae being sister to Phrymeae + Diplaceae + Leucocarpeae, with the relationship among the latter three showing extensive gene tree discordance. However, no individual reticulation event among the Phrymaceae tribes was strongly supported. Instead, we found evidence for introgression from closely related species to *Erythranthe bicolor* (Hartw. ex Benth.) G.L. Nesom & N.S. Fraga. Our analyses did not identify any ancient WGD events in Phrymaceae; instead, small‐scale gene duplications involved in defense, stress response, growth and development, and certain biochemical pathways are candidates for potential drivers that underlie macroevolutionary diversification of Phrymaceae.

## MATERIALS AND METHODS

### Taxon sampling

Transcriptomes were newly generated from eight accessions representing seven ingroup Phrymaceae species for this study (Appendix S1). Seeds were collected from natural populations and were cold‐treated in soil for a week at 4°C in the dark before growing with 15‐hour daylight in a greenhouse. Young leaves and flower buds were flash frozen in liquid nitrogen. RNA extraction, library preparation (rRNA removal or poly‐A enrichment), and sequencing procedures are detailed in Appendix S1. In addition, we included three genomes and five transcriptomes from Phrymaceae that are publicly available (Appendix S2). Together our sampling included 16 accessions representing 15 Phrymaceae species in four of the five tribes (missing the tropical Asian tribe Cyrtandromoeeae) and five of the 14 genera (Barker et al., 2012; Liu et al., 2020). We also included four genomes and four transcriptomes in closely related Lamiales families (Zhang et al., 2020).

### Data processing for nuclear genes

Read processing, assembly, and translation were carried out following Morales‐Briones et al. (2021). Homology inference started with an all‐by‐all BLASTN search on coding sequences (CDS) with an *E* value cutoff of 10. Hits were filtered with a minimal hit coverage of 40%. Homolog groups were clustered using MCL version 14‐137 (van Dongen, 2000) with a minimum minus log‐transformed *E* value cutoff of 5 and an inflation value of 1.4. Only clusters with at least 20 out of 24 taxa represented were retained. Sequences from each cluster were aligned using the OMM_MACSE pipeline version 10.02 (Scornavacca et al., 2019), which pre‐filters non‐homologous sequence fragments with HMMCleaner (Di Franco et al., 2019) before translation accounting for frameshifts using MACSE version 2.03 (Ranwez et al., 2018). The resulting CDS alignments were trimmed to remove columns with more than 90% missing data using Phyx (Brown et al., 2017). Homolog trees were built with RAxML version 8.2.11 (Stamatakis, 2014) using the GTRCAT model and 200 rapid bootstrap (BS) replicates. Sequences from the same taxon that were monophyletic or paraphyletic were removed, keeping only the sequence with the highest number of characters in the trimmed alignment. Spurious sequences forming long branches on gene trees were detected and removed with TreeShrink version 1.3.2 (Mai and Mirarab, 2018) with the ʻper‐gene’ mode and a false positive error rate threshold (α) of 0.001. The resulting trees were visually inspected, and deep paralogs producing internal branch lengths longer than 0.25 were cut apart, retaining subclades with at least 20 taxa to obtain final homolog trees.

Orthology inference was carried out using the “monophyletic outgroup” approach and the script “prune_paralogs_MO.py” from Yang and Smith (2014). The approach filters unrooted homolog trees, requiring outgroups to be single‐copy and monophyletic. It then roots each homolog tree by the outgroups, traverses the ingroups from root to tip and removes the side with fewer taxa each time a gene duplication event is detected, until every taxon is represented by a single sequence. We set the three Lamiaceae genomes as outgroups (Zhang et al., 2020), keeping only ortholog groups with at least 15 taxa for subsequent analyses.

### Species tree inference and evaluation of support

Sequences from individual ortholog groups were aligned using OMM_MACSE. Columns with more than 20% missing data were trimmed with Phyx, and only alignments with at least 1000 characters and all 24 taxa were retained. We first estimated a maximum likelihood (ML) tree of the concatenated matrix with IQ‐TREE version 2.1.13 (Minh et al., 2020) searching for the best partition scheme using ModelFinder implemented within IQ‐TREE (Lanfear et al., 2012), followed by 100 searches for the best ML tree inference and 1000 ultrafast bootstrap replicates. To estimate a coalescent‐based species tree, we first inferred individual gene trees with IQ‐TREE using extended model selection (Kalyaanamoorthy et al., 2017) followed by 100 searches for the best ML tree and 200 non‐parametric bootstrap replicates for clade support. Gene trees were then used to infer a species tree with ASTRAL‐III version 5.6.3 (Zhang et al., 2018) using local posterior probabilities (LPP; Sayyari and Mirarab, 2016) to assess clade support.

To explore discordance among gene trees, we calculated the number of concordant and discordant bipartitions on each node of the species tree using PhyParts (Smith et al., 2015). We mapped bipartitions from gene trees with bipartition BS support of at least 50% against the IQ‐TREE tree from the concatenated supermatrix (identical to the ASTRAL topology; see Results). Next, to distinguish conflict from poorly supported branches, we carried out a Quartet Sampling (QS; Pease et al., 2018) analysis using the concatenated supermatrix, the IQ‐TREE tree, and 1000 replicates. Lastly, to visualize gene tree conflict, we built a cloudogram using the DensiTree function of phangorn version 2.7.1 (Schliep et al., 2017). Individual orthologous gene trees were time‐calibrated with TreePL version 1.0 (Smith and O'Meara, 2012) for the sole purpose of visualization using cloudogram: the root was fixed to 70.3 MYA, the most recent common ancestor (MRCA) of Lamiaceae was fixed to 57.69 MYA, and the MRCA of all remaining species was fixed to 68.8 MYA (Zhang et al., 2020).

### Plastome assembly and tree inference

We obtained nine reference plastomes from RefSeq (Appendix S3). For the remaining species, we assembled the plastomes from either transcriptomic or genomic reads (Appendix S3) with Fast‐Plast version 1.2.8 (McKain and Wilson, 2017). In four cases, those assemblies resulted in low plastome coverage and were redone using alternative transcriptomic or genomic libraries (Appendix S3). When the resulting plastomes were incomplete (7 out of 14 accessions), filtered contigs from Spades version 3.9.0 (Bankevich et al., 2012) were mapped to the closest available reference plastome using Geneious version 11.1.5 (Kearse et al., 2012) to produce oriented and contiguous contigs with missing regions masked with ‘N’. The assembly of *Striga asiatica* (L.) Kuntze (Orobanchaceae) had many contigs that were poorly mapped even to congeneric plastomes, likely due to major structural rearrangements in this hemiparasitic species (Frailey et al., 2018). We replaced it with the published plastome of *Striga forbesii* Benth. (Appendix S3) for downstream analyses.

The resulting plastomes with one inverted repeat removed were aligned with MAFFT and columns with more than 50% missing data were trimmed with Phyx. An ML tree was inferred with IQ‐TREE with automated extended model selection, 100 searches for the best ML tree and 1000 rapid BS replicates. Additionally, we used QS with 1000 replicates to evaluate branch support.

### Tests for reticulate evolution

We investigated two regions on the nuclear species tree with elevated gene tree conflict: (1) the backbone of Phrymaceae using one species for each well‐supported clade corresponding to a tribe; and (2) among *Erythranthe cardinalis* (Douglas ex Benth.) Spach, *E. lewisii*, and *E. bicolor*.

For each of the two regions, we first ran PhyParts using all taxa from the reduced dataset. We then removed one taxon at a time to determine which taxon produced the highest gene tree conflict. We inferred species networks using ML (Yu et al., 2014) in PhyloNet version 3.6.9 (Than et al., 2008) with the command “InferNetworks_ML” from individual ML gene trees. Network searches were performed allowing for up to three reticulation events and optimizing the branch lengths and inheritance probabilities of the inferred species networks. To estimate the optimal number of reticulations and to test whether a species network fits our gene trees better than a strictly bifurcating tree, we computed the likelihood scores of the nuclear and plastid trees given the individual gene trees using the command ‘CalGTProb’ (Yu et al., 2012). We performed model selection using the Akaike information criterion (AIC; Akaike, 1973), bias‐corrected AIC (AIC_*c*_; Sugiura, 1978) and the Bayesian information criterion (BIC; Schwarz, 1978). Next, we performed a more thorough PhyloNet analysis using a Bayesian inference of species networks approach (Wen et al., 2016) with the command “MCMC_GT”, full likelihood, and allowing up to three reticulation events. Analyses consisted of four independent runs with four reversible‐jump Markov chain Monte Carlo (RJMCMC) chains, temperatures set to one cold and two hot chains (1.0, 2.0, and 3.0 respectively), 30 million generations, sampling every 1000 generation, and a burn‐in of 500,000 generations. The four MCMC runs were summarized with the command “MCMC_GT ‐sum” to produce a maximum posterior probability (MPP) network. Convergence was assessed once the posterior sampling reached ESS (effective sample size) ≥ 200.

In addition to PhyloNet, we also tested for hybridization with HyDe (Blischak et al., 2018), which uses site pattern frequencies (Kubatko and Chifman, 2019) to quantify admixture (γ) between two parental lineages that form a hybrid lineage. We tested all triplet combinations in all directions using ‘run_hyde.py’, the concatenated nuclear alignment, and a mapping file to assign individuals to species. Test significance was assessed with a Bonferroni correction (α = 0.05) for the number of tests conducted with estimates of γ between 0 and 1 (Blischak et al., 2018).

### Gene and whole genome duplication events

We employed three approaches (Yang et al., 2018) to detect WGD events in Phrymaceae: (1) We summarized chromosome counts from Nesom (2012) and the Chromosome Counts Database (Rice et al., 2015); (2) We mapped gene duplication events onto the nuclear species tree by extracting rooted ingroup clades from the final homolog trees with an average BS ≥ 50 and at least 15 taxa. Gene duplication events were then mapped onto the MRCA on the species tree when two or more taxa overlapped between the two daughter clades on the rooted ingroup clade (“extract_clades.py” and “map_dups_mrca.py” from website https://bitbucket.org/blackrim/clustering); and (3) We analyzed the distribution of synonymous distances (Ks) from RNA‐seq (website https://bitbucket.org/blackrim/clustering; “ks_plots.py”). Ks peaks were identified using a mixture model in mixtools version 1.2.0 (Benaglia et al., 2009).

To identify genes with elevated instances of gene duplication within Phrymaceae, we extracted Phrymaceae clades from the final homologs. We then obtained functional annotation for the ten Phrymaceae clades with the highest number of sequences using the *Erythranthe guttata* genome annotation (Hellsten et al., 2013).

## RESULTS

### Sequence processing

Organellar reads represented 30 to 57% of quality‐filtered read pairs in RNA‐seq libraries prepared using rRNA removal, compared to 0.13 to 0.3% in libraries prepared using Poly‐A enrichment (Appendix S1). Of the eight newly generated transcriptomes, we retained 13.7 to 21.4 million nuclear read pairs after quality filtering and separating organellar reads; each CDS set represented 49 to 62% of nuclear genes when compared against the *Erythranthe guttata* reference genome (Appendix S2). Although libraries prepared by rRNA removal produced lower numbers of nuclear reads, they produced more contiguous assemblies and some of the highest numbers of genes in our final nuclear ortholog set (Appendix S2). This can be due to ribosomal depletion resulting in more even read coverage in slightly degraded RNA samples compared to poly‐A enrichment.

Assemblies from each of the six genomic libraries produced full plastomes in a single contig (Appendix S3). Despite the large numbers of plastid reads from the six libraries prepared by rRNA removal, only one complete plastome was assembled due to uneven coverage. Still, libraries prepared by rRNA removal produced contiguous contigs that covered most of the plastomes and recovered similar numbers of CDS compared to the full plastomes (Appendix S3).

### Orthology inference and phylogenetic analysis

**Figure 2:**
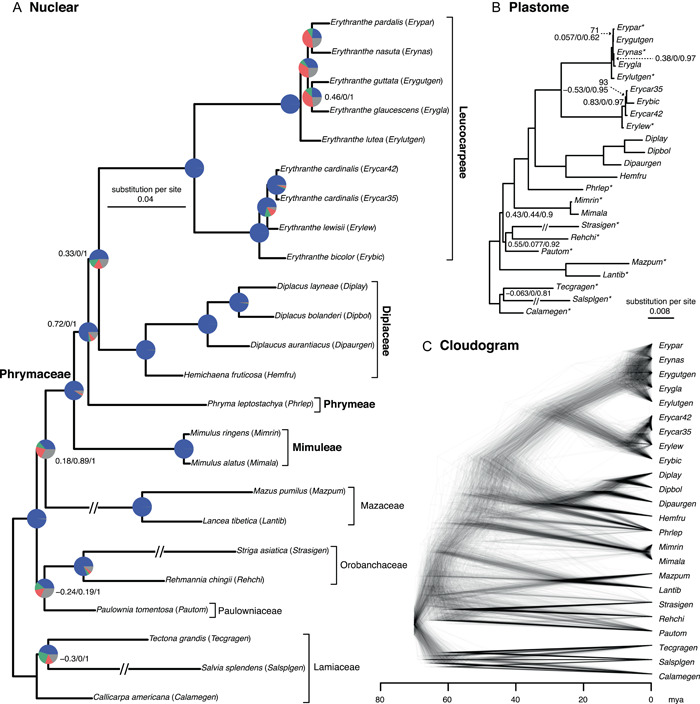
(A) Maximum likelihood phylogeny of Phrymaceae inferred with IQ‐TREE from the concatenated 732‐nuclear gene supermatrix. Quartet Sampling (QS) scores are shown next to nodes, except those with maximum QS support (1/–/1). QS scores: Quartet concordance/Quartet differential/Quartet informativeness. All nodes have maximum bootstrap support (BS = 100) and local posterior probability (LLP = 1). Pie charts represent the proportion of ortholog trees that support that clade (blue), the main alternative bifurcation (green), the remaining alternatives (red), and the remaining alternatives with (conflict or support) < 50% bootstrap support (gray). Branch lengths as number of substitutions per site (scale bar). Exceptionally long branches are shortened with a broken segment (//) for illustration purposes (see Appendix S4 for original branch lengths); (B) Maximum likelihood phylogeny inferred with IQ‐TREE from plastomes. Bootstrap support is shown above branches and QS scores below the branches. Maximum BS and QS support values are not shown. Branch lengths as number of substitutions per site (scale bar). Longest branches are shortened with a broken segment (//) for illustration purposes (See Appendix S4 for original branch lengths); (C) Cloudogram inferred from 732 nuclear ortholog trees. Scale in millions of years ago (mya).

The final set of nuclear orthologs included 732 genes, and the concatenated matrix consisted of 1,246,075 aligned columns with a character occupancy of 93.1% (Appendix S2). The topologies from the IQ‐TREE and ASTRAL trees were identical and all nodes had maximum support (BS = 100, LPP = 100; Figures 1, 2A; Appendix S4). The monophyly of Phrymaceae and each tribe of Phrymaceae were strongly supported by almost all informative gene trees (BS > 50; blue in Figure 2A and Appendix S5) and full QS support (1/–/1; i.e., all sampled quartets supported that branch). The sister relationship of Leucocarpeae and Diplaceae was supported by 359/564 informative gene trees and strong Quartet Concordance (QC = 0.3), but the Quartet Differential (QD = 0) indicates the presence of a single alternative topology (Diplaceae sister to Phrymeae). Similarly, the placement of Phrymeae as sister to Leucoparpeae and Diplaceae was supported by 525/601 informative gene trees, strong QC (0.72) and signal of a single alternative topology (QD = 0; Mimulaeae sister to Phrymeae). This is consistent with the cloudogram (Figure 2C) that showed discordance in the backbone of Phrymaceae, especially on the placement of Phrymeae (“Phrlep”).

The final cpDNA alignment included 128,056 characters with a character occupancy of 89%. The plastome phylogeny recovered the monophyly of Phrymaceae and each of its tribes with maximum support (BS = 100, QS 1/–/1; Figure 2B) and the backbone relationships were identical to the nuclear results (Figure 2A; Appendix S5). However, relationships among closely related Phrymaceae taxa differ in two places (Figure 2; A vs. B): (1) The two *Erythranthe cardinalis* accessions were sister to each other in the nuclear tree but were paraphyletic with *E. bicolor* nested among them in the cpDNA tree; (2) relationships among *Erythranthe pardalis* (Pennell) G.L. Nesom, *E. nasuta* (Greene) G.L. Nesom, *E. guttata*, and *E. glaucescens* (Greene) G.L. Nesom showed extensive gene tree conflict among nuclear genes, low quartet concordance and dominant secondary topologies in the cpDNA tree, and conflicting topology between cpDNA and nuclear trees. In addition, extensive nuclear gene tree conflict and discordance between nuclear and cpDNA trees are present among other Lamiales families sampled.

### Phylogenetic network analyses

**Figure 3:**
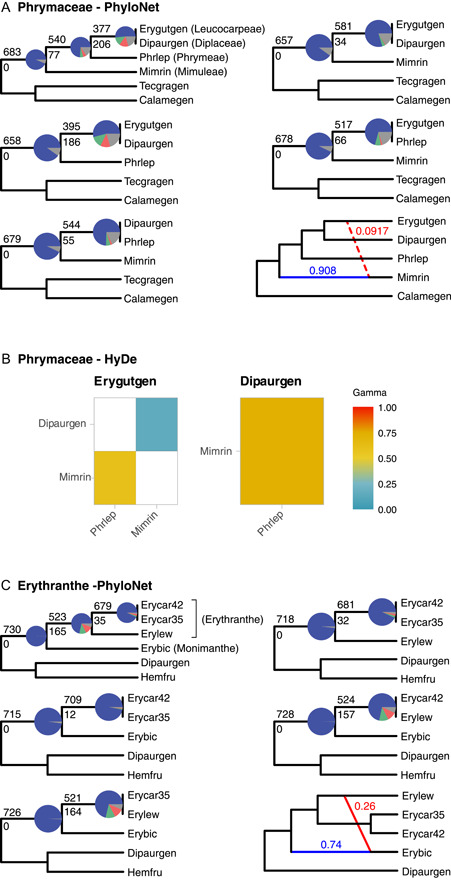
Gene tree conflict, phylogenetic network, and tests for hybridization events using reduced taxon sampling. (A) Phylogenetic network for the Phrymaceae backbone recovered from PhyloNet, with one representative species for each tribe. (B) Significant hybridization events among the Phrymaceae backbone recovered from HyDe analyses. (C) Phylogenetic network for *Erythranthe cardinalis*, *E. lewisii*, and *E. bicolor* recovered from PhyloNet. Cladograms (A and C) showing relationships among the reduced taxon set, removing one tip at a time, and maximum posterior probability (MPP) network from the PhyloNet Bayesian inference. Pie charts on cladograms represent the proportion of gene trees that support that clade (blue), the main alternative bipartition (green), the remaining alternatives (red), and conflict or support with <50% bootstrap support (gray). Numbers above and below branches represent the number of concordant and discordant gene trees, respectively. Red and blue branches in networks indicate the minor and major edges, respectively, of hybrid nodes, with the inheritance probabilities next to each branch. The dotted edge indicates uncertainty in inference (see Results and Discussion). HyDe matrices (B) denote parental lineage 1 (P1) on the x‐axis and parental lineage 2 (P2) y‐axis. Only colored boxes denote possible combinations of P1 and P2 as parents of hybrid species. The color scale represents the value of the admixture parameter γ for each hybridization event. Recent 50:50 hybrids would have a γ ~0.5. Values of γ approaching 0 indicate a major hybrid contribution from P1, and values approaching 1 indicate a major hybrid contribution from P2, with both cases representing back crossing. HyDe tests did not recover any significant hybridization event among *Erythranthe cardinalis*, *E. lewisii*, and *E. bicolor*. For taxon abbreviations, see Figure 2.

Phylogenetic network analyses focused on two ingroup areas with elevated levels of conflict. To investigate the backbone of Phrymaceae, we used one taxon to represent each tribe. The cloudogram (Figure 2C) showed *Phryma leptostachya* L. (Phrymeae) shifted its placement among other Phrymaceae tribes. When removing one tip at a time, conflict among nuclear gene trees (red and green in Figure 3A) reduced the most when removing *Phryma*, followed by removing *Erythranthe guttata* (Leucocarpeae). Visual inspection of individual gene trees confirmed that *Phryma*'s placement shifted among genes, with short internal branches attached to the backbone of Phrymaceae. PhyloNet ML searches (Appendix S6) recovered three networks with small amounts of gene flow towards *Mimulus ringens* L. Model selection (Appendix S7) using AIC and AIC_*c*_ both preferred three reticulations while BIC did not support significant differences among the three networks. The MPP network from the MCMC PhyloNet searches (Figure 3A) recovered the same network as the 1‐reticulation network from ML and estimated that 9.17% of *M. ringens* genes had contribution from *E. guttata*, with the 95% credible set consisting of a single network. HyDe (Figure 3B; Appendix S8) analyses recovered *E. guttata* received parental contributions from Mimuleae, Phrymeae, and Leucocarpeae; and *Diplacus aurantiacus* (Curtis) Jeps. received parental contributions from Mimuleae and Phrymeae. Given the disagreement between PhyloNet and HyDe analyses, none of the putative hybridization events were well‐supported. As almost all informative gene trees supported the monophyly of each Phrymaceae tribe in our taxon sampling (except that Phrymeae was represented by only one sample), any potential hybridization events would have occurred among stem branches of tribes in our taxon sampling, and additional tests using different taxa to represent each tribe are unlikely to change PhyloNet or HyDe results.

The second instance of conflict we focused on was among *Erythranthe bicolor, E. cardinalis* (two accessions), and *E. lewisii*. Consistent with the cloudogram (Figure 2C) where some gene trees supported *E. bicolor* being sister to *E. lewisii*, removing either *E. bicolor* or *E. lewisii* (Figure 3C) removed most of the gene tree conflicts. PhyloNet ML analysis (Appendix S6) recovered networks with 38 to 48% of *E. bicolor* genes from *E. lewisii* or its close relatives. AIC or AIC_*c*_ did not prefer any network, while BIC preferred the 1‐reticulation network (Appendix S7). PhyloNet MCMC searches recovered a 95% credible set of three 1‐reticulation networks with gene flow towards *E. bicolor*, but the source of gene flow varied among networks (Appendix S9). The MPP network (54% of the credible set) showed that *E. bicolor* had 26% genes from *E. lewisii*, similar to the 1‐reticulation ML network (Figure 3B). HyDe, on the other hand, did not identify any significant hybridization events.

A third area with elevated gene tree conflict was among *Erythranthe pardalis*, *E. nasuta*, *E. guttata*, and *E. glaucescens*. However, as branches among them were short and we lacked any intraspecific sampling, we did not carry out additional analyses on reticulate evolution.

### Gene and genome duplications

Mapping gene duplication events did not reveal any node with more than 4.3% of gene duplications in Phrymaceae (Appendix S10). Similarly, the Ks plots (Appendix S11) did not support any Phrymaceae‐specific WGD that occurred in the common ancestry of more than one taxon sampled. All 24 transcriptomes and genomes included in this study shared two optimal mixing components (i.e., Ks peaks). The first component had a Ks mean of 1.8 to 2.2, corresponding to a whole‐genome triplication event early in the core eudicots (Jiao et al., 2012). The second component had Ks means of 0.3 to 0.9 (lower in woody species and higher in herbaceous species), corresponding to a WGD at the MRCA of the core Lamiales (Zhang et al., 2020). A third component at Ks~0.1 was found only in *Erythranthe lutea* (L.) G.L. Nesom, corresponding to a previously reported WGD event (Edger et al., 2018). In addition, *Diplacus layneae* (Greene) G.L. Nesom and *Erythranthe guttata* each showed a putative Ks peak at ~0.04 and 0.09 respectively. However, chromosome counts (Appendix S10) did not support a WGD in either species. Overall, all sampled Phrymaceae species except *Erythranthe lutea* had low chromosome counts compared to outgroups, and the uptick in Ks density below 0.1 was likely due to recent small‐scale duplications or artifacts from de novo transcriptome assembly. Among non‐Phrymaceae species, *Salvia splendens* Sellow ex J.A. Schultes showed a Ks peak ~0.1, consistent with a WGD in *Salvia* and relatives within Lamiaceae (Godden et al., 2019). Both *Mazus pumilus* (Burm.f.) Steenis and *Lancea tibetica* (Hook.f.) Thomson showed a Ks peak ~0.07, which could be due to WGD or small‐scale duplications.

In addition to WGD events, we investigated gene family expansion for evidence of genes that may have contributed to macroevolutionary diversification in Phrymaceae. The ten Phrymaceae genes (Appendix S12) with the highest numbers of copies in the final homolog trees were involved in defense/immune response (Serine protease inhibitor, aspartyl protease, MLP‐like protein), stress response (HSP20‐like chaperones, Ribosomal protein L10 family protein), mitochondria organization (prohibitin 2), regulating plant growth (small auxin up‐regulated RNA‐like auxin‐responsive protein family), cell wall architecture (Glycosyl hydrolase), and various other biochemical processes (S‐adenosyl‐L‐methionine‐dependent methyltransferases, hydroxymethyltransferase 4). Since we reduced sequences from the same sample that formed monophyletic or paraphyletic relationships, we effectively excluded isoforms from alternative splicing, assembly artifacts, and recent copy number increase involving only a single sample, as these are difficult to quantify using de novo assembled transcriptomes. Therefore, only gene duplication events involving more than one taxon in our sampling contributed to our copy number counts. Given our much denser taxon sampling in Leucocarpeae and Diplaceae, the top ten are heavily influenced by genes that had multiple rounds of gene duplications in these two tribes.

## DISCUSSION

### Extensive gene tree discordance and potential hybridization events in Phrymaceae

Our phylogenomic analyses recovered strong support of the monophyly of Phrymaceae and each of its tribes sampled. We also recovered extensive and well‐supported gene tree discordance along the backbone of Phrymaceae. The discordance is not an artifact of gene and genome duplications; nor is any particular reticulation event well‐supported by phylogenetic network analyses and hypothesis testing. Therefore, phylogenetic uncertainty, ILS, population structure, and to a smaller extent analytical errors (assembly, orthology inference, and gene tree estimation) likely contributed to the extensive gene tree discordance among Phrymaceae tribes.

Among closely related species, although HyDe did not recover any significant hybridization event, our phylogenetic network analyses support introgression from *E. lewisii* or close relatives towards *E. bicolor*. Plastome data (Figure 2B) recovered *E. bicolor* being nested among accessions of *E. cardinalis*, suggesting that *E. cardinalis* is also involved in the reticulation. However, without sampling of other closely related species or additional within‐species sampling, the timing, source, and prevalence of the introgression is unclear (Tricou et al., 2022). Nelson et al. (2021) analyzed over 8,000 nuclear gene trees and identified extensive reticulation among *E. lewisii*, *E. cardinalis*, and *E. parishii* (Greene) G.L. Nesom & N.S. Fraga (not sampled in our study), with *E. bicolor* set as their outgroup. Our analyses suggest that *E. bicolor* or its close relatives are involved in introgression with *E. lewisii*, *E. cardinalis*, and/or other close relatives. Therefore *E. bicolor* may not be assumed as the outgroup for introgression analyses involving *E. lewisii* and *E. cardinalis*.

In summary, our phylogenetic analyses suggest that: (1) network inferences are sensitive to the methods used, sources of data, and taxon sampling, including the choice of both ingroups and outgroups; (2) Despite phylogenetic uncertainty along the Phrymaceae backbone, most gene trees support Mimuleae (likely together with the unsampled Cyrtandromoeeae) being sister to a strongly supported clade of Phrymeae + Diplaceae + Leucocarpeae, consistent with certain previous Sanger‐based studies (as summarized in Barker et al. [2012]; yellow topologies in Figure 1); and (3) Our results reject the monophyly of the monkeyflower genus *Mimulus* s.l. (= part of Mimuleae + part of Leucocarpeae + part of Diplaceae).

### Genomic drivers of macroevolution in Phrymaceae

Analyses of chromosome counts, gene tree mapping, and Ks plots did not find any evidence for WGD in Phrymaceae that occurred in the common ancestor of more than one taxon sampled. However, as we did not sample any members of *Erythranthe* sect. *Mimulosma* or sect. *Paradantha* with *n* = 16, it is unclear whether those show WGD relative to *Erythranthe* lineages with *n* = 8 (Beardsley et al., 2004; Barker et al. 2012). Our results are consistent with the previous analyses using linkage maps to compare *E. lewisii* and *E. guttata* (Fishman et al., 2014) and using whole genome sequences of *E. guttata* and *E. lutea* (Edger et al., 2018), both primarily focused on species in the tribe Leucocarpeae. Our analysis broadened the genome‐wide sampling to four of the five tribes in Phrymaceae and found that ancient WGD is not a driving force in macroevolution of Phrymaceae. Instead, reticulate evolution, small‐scale duplication in genes involved in defense, stress response, growth and development, and certain biochemical pathways are among potential drivers of macroevolutionary diversification in Phrymaceae. Our study provides initial insights into the gene space of species across Phrymaceae and potential genomic drivers of macroevolution in the family. In addition, our newly generated transcriptome datasets by rRNA removal provide data for future studies looking into non‐coding RNAs.

## CONCLUSIONS

Our phylogenomic analysis evaluated the support (or the lack of ) in the backbone of Phrymaceae, confirmed the polyphyly of *Mimulus* s.l., and detected an area of reticulate evolution among closely related species. We show that analysis of reticulate evolution is sensitive to taxon sampling and methods used. We also show a lack of ancient WGD events in Phrymaceae; instead, small‐scale duplications are potential drivers that underlie macroevolutionary diversification of Phrymaceae.

Our analyses demonstrate that genome‐scale data do not always “resolve” phylogenetic relationships. Instead, they provide resolution for some areas, but also recover “clouds” and “networks” that point to future opportunities for investigating their significance in adaptation and lineage diversification.

## AUTHOR CONTRIBUTIONS

Y.Y. and D.L.G. designed the study; Y.H., D.L.G., J.M.S., C.D.G., and Y.Y. generated the data; D.F.M.‐B., N.L., and Y.Y. analyzed data and drafted the manuscript. All authors contributed to the writing and approved the final version.

## Supporting information


**Appendix S1.** Collection, plant growth, and sequencing information for the eight newly generated transcriptomes.

**Appendix S2.** Taxon sampling, source of data, and nuclear matrix statistics. Naming authorities above species level (Stevens, 2001 onwards): (1) Order: Lamiales Bromhead; (2) Lamiales families: Phrymaceae Schauer, Orobanchaceae Ventenat, Mazaceae Reveal, and Paulowniaceae Nakai; (3) Phrymaceae tribes: Diplaceae Bo Li, B. Liu, S. Liu & Y. H. Tan; Phrymeae Hogg; Leucocarpeae Conzatti; Mimuleae Dumortier; Cyrtandromoeeae Bo Li, B. Liu, S. Liu & Y. H. Tan; and (4) Phrymaceae genera: *Diplacus* Nuttall, *Hemichaena* Bentham, *Erythranthe* Spach, *Mimulus* L., and *Phryma* L.

**Appendix S3.** Sources of plastome data and assembly statistics.

**Appendix S4.** (A) Maximum likelihood phylogeny of Phrymaceae inferred with IQ‐TREE from the concatenated 732‐nuclear gene supermatrix. Numbers above branches represent bootstrap support (BS). Branch lengths as number of substitutions per site (scale bar). (B) ASTRAL tree of Phrymaceae inferred from the 732 nuclear gene trees. Local posterior probabilities (LLP) are shown next to nodes. Internal branch lengths are in coalescent units (scale bar). (C) Maximum likelihood phylogeny of Phrymaceae inferred with IQ‐TREE from plastomes. BS values are shown above branches. Branch lengths as number of substitutions per site (scale bar).

**Appendix S5.** Maximum likelihood cladogram of Phrymaceae inferred with IQ‐TREE from the concatenated 732‐nuclear gene supermatrix. Pie charts represent the proportion of gene trees that support that clade (blue), the main alternative bifurcation (green), the remaining alternatives (red), and conflict or support that have <50% bootstrap support (gray). Number above and below branches represent the number of concordant and discordant informative gene trees, respectively.

**Appendix S6.** Species network inferred from PhyloNet maximum likelihood analyses with one to three maximum reticulations of the reduced data sets. (A) Phrymaceae backbone. (B) *Erythranthe cardinalis*, *E. lewisii*, and *E. bicolor*. Red and blue branches indicate the minor and major edges, respectively, of hybrid nodes. Numbers next to colored branches indicate inheritance probabilities for each hybrid node.

**Appendix S7.** Model testing between trees and PhyloNet networks for the reduced data sets of Phrymaceae and *Erythranthe*. The number of parameters for each test was set to equal the number of branch lengths plus the number of inheritance probabilities. The number of gene trees was used to correct for finite sample size.

**Appendix S8.** HyDe tests for hybridization events along the backbone of Phrymaceae.

**Appendix S9.** 95% credibility set for the reduced taxon set of *Erythranthe cardinalis*, *E. lewisii*, and *E. bicolor* from Bayesian inference in PhyloNet. (A) The maximum posterior probability (MPP) network representing 54% of the credibility set. (B) The second most frequent network (30%). (C) The third most frequent network (13.5%). Red and blue branches indicate the minor and major edges, respectively, of hybrid nodes. Numbers next to colored branches indicate inheritance probabilities for each hybrid node.

**Appendix S10.** Maximum likelihood cladogram of Phrymaceae inferred with IQ‐TREE from the concatenated 732‐nuclear gene supermatrix. Numbers above branches are gene duplication counts and numbers below branches are gene duplication percentages. Numbers next to species names are haploid chromosome numbers. All chromosome counts are from the Chromosome Counts Database (Rice et al., 2015), except *Erythranthe pardalis* (Nesom, 2012). When multiple independent counts gave a single consistent chromosome number but different counts were each reported by a single study, we ignored the outlier numbers. Inset: Histogram of percentages of gene duplication per branch.

**Appendix S11.** Distribution of synonymous distance among gene pairs (Ks) for each genome or transcriptome. (A) Distribution of raw Ks values between 0 and 3. (B) Distribution of Ks values zooming in to between 0 and 0.5. (C) Plots of log‐transformed Ks values. Colored lines indicate components inferred using a mixture model. Blue lines indicate a component from an ancestral whole genome triplication event early in core eudicots; red lines are from more recent whole genome or small‐scale duplication events.

**Appendix S12.** Phrymaceae clades extracted from the final homologs with the highest number of sequences.

## Data Availability

Raw reads of newly sequenced transcriptomes were deposited in the NCBI Sequence Read Archive (BioProject: PRJNA770153). Analysis files are available from the Dryad Digital Repository at https://doi.org/10.5061/dryad.83bk3j9t6 (Morales‐Briones et al., 2022.).
