# Supplementary material for: Phylogenomic analyses in Phrymaceae reveal extensive gene tree discordance in relationships among major clades
Source: Am J Bot. 2022 Jun 5;109(6):1035–46. doi: 10.1002/ajb2.1860 (PMC9328367; doi:10.1002/ajb2.1860)
Supplement: Supplementary file 4 — Appendix S4. (A) Maximum likelihood phylogeny of Phrymaceae inferred with IQ‐TREE from the concatenated 732‐nuclear gene supermatrix. Numbers above branches represent bootstrap support (BS). Branch lengths as number of substitutions per site (scale bar). (B) ASTRAL tree of Phrymaceae inferred from the 732 nuclear gene trees. Local posterior probabilities (LLP) are shown next to nodes. Internal branch lengths are in coalescent units (scale bar). (C) Maximum likelihood phylogeny of Phrymaceae inferred with IQ‐TREE from plastomes. BS values are shown above branches. Branch lengths as number of substitutions per site (scale bar). [file AJB2-109-1035-s004.pdf]

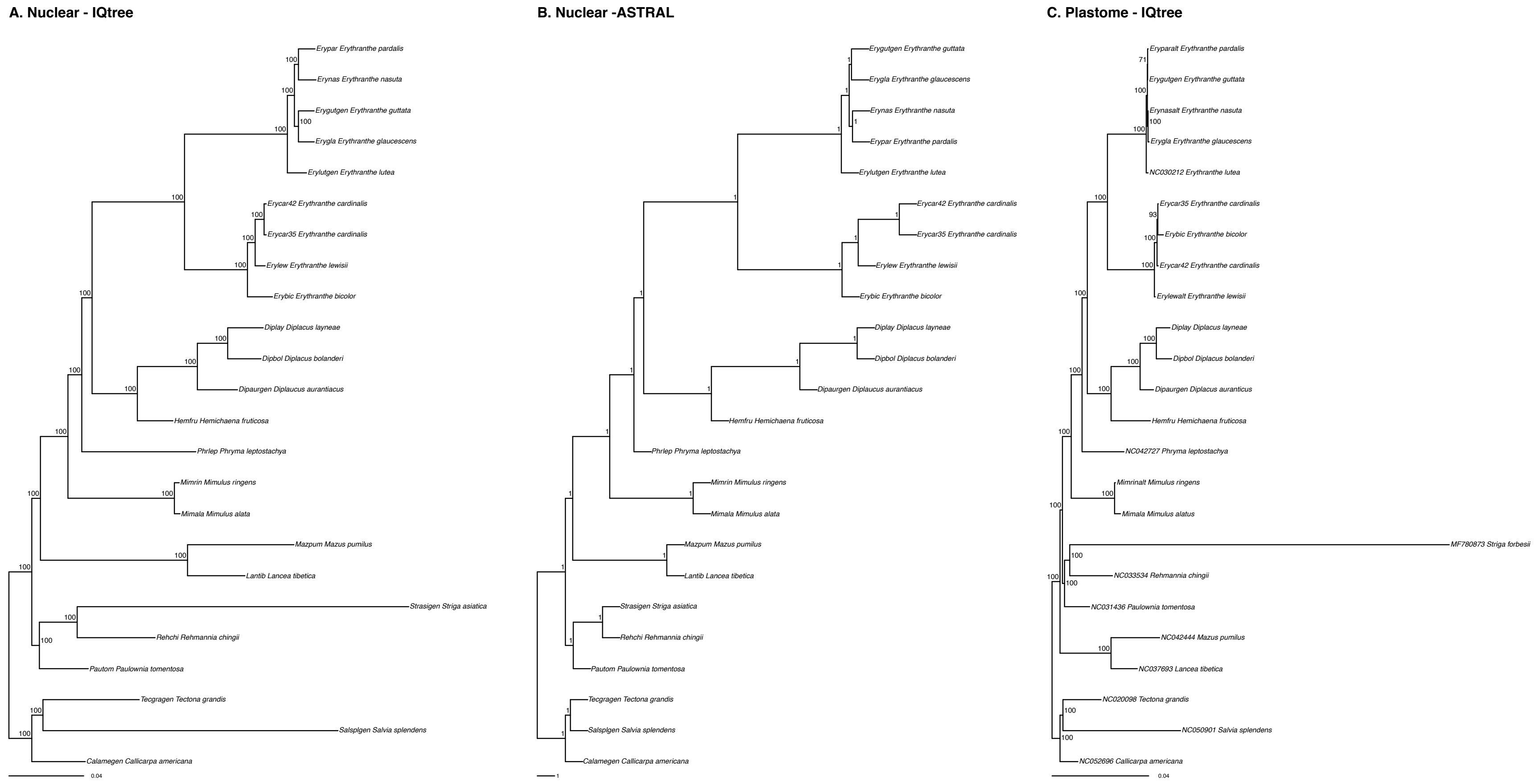

Appendix S4. A. Maximum likelihood phylogeny of Phrymaceae inferred with IQ-TREE from the concatenated 732-nuclear gene supermatrix. Numbers above branches represent bootstrap support (BS). Branch lengths as number of substitutions per site (scale bar on the bottom). B. ASTRAL tree of Phrymaceae inferred from the 732 nuclear gene trees. Local posterior probabilities (LLP) are shown next to nodes. Internal branch lengths are in coalescent units (scale bar on the bottom). C. Maximum likelihood phylogeny of Phrymaceae inferred with IQ-TREE from plastomes. BS values are shown above branches. Branch lengths as number of substitutions per site (scale bar on the bottom).
