# Supplementary material for: Phylogenomic analyses in Phrymaceae reveal extensive gene tree discordance in relationships among major clades
Source: Am J Bot. 2022 Jun 5;109(6):1035–46. doi: 10.1002/ajb2.1860 (PMC9328367; doi:10.1002/ajb2.1860)
Supplement: Supplementary file 5 — Appendix S5. Maximum likelihood cladogram of Phrymaceae inferred with IQ‐TREE from the concatenated 732‐nuclear gene supermatrix. Pie charts represent the proportion of gene trees that support that clade (blue), the main alternative bifurcation (green), the remaining alternatives (red), and conflict or support that have <50% bootstrap support (gray). Number above and below branches represent the number of concordant and discordant informative gene trees, respectively. [file AJB2-109-1035-s012.pdf]

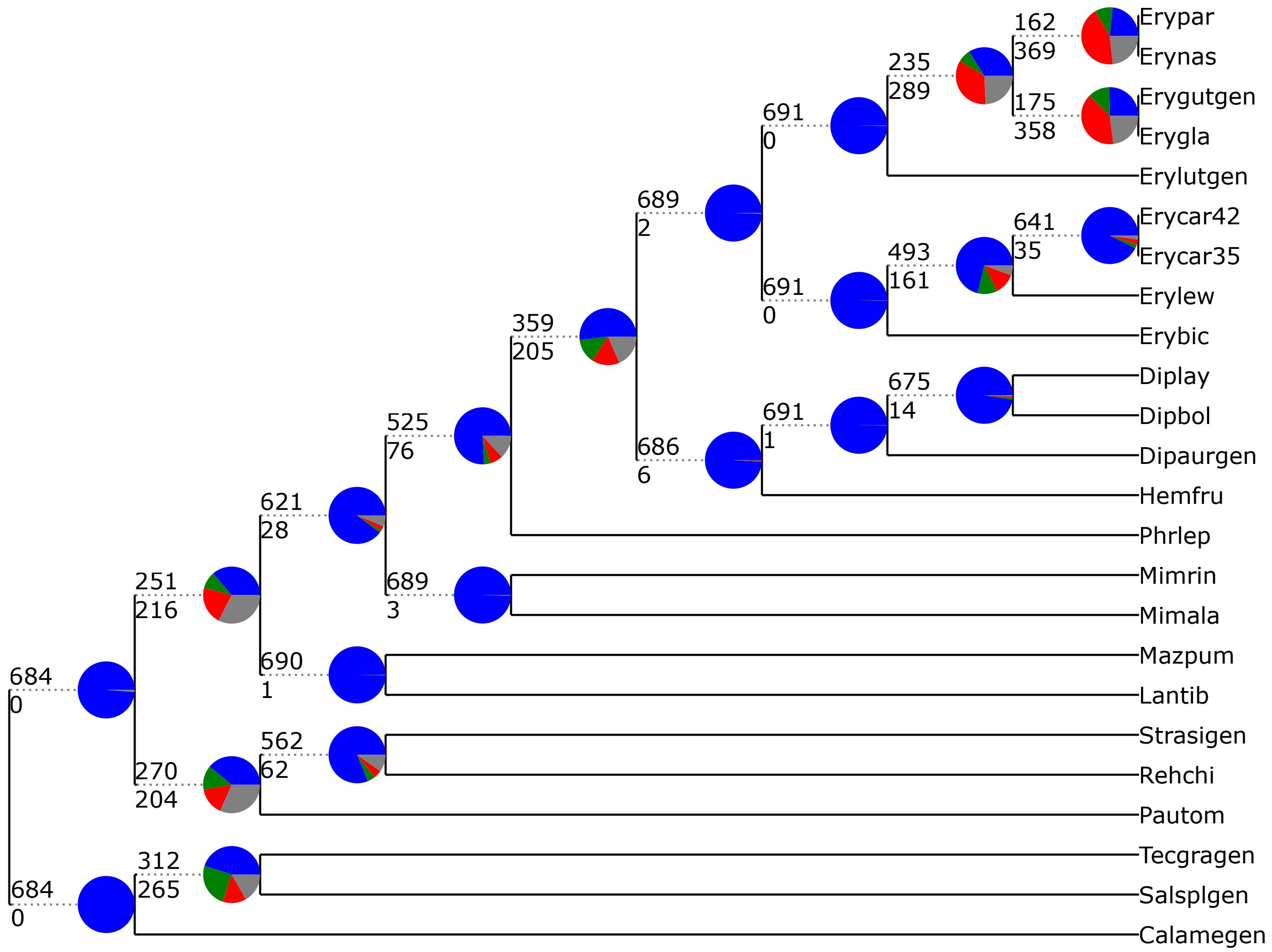

Appendix S5. Maximum likelihood cladogram of Phrymaceae inferred with IQ-TREE from the concatenated 732-nuclear gene supermatrix. Pie charts represent the proportion of gene trees that support that clade (blue), the main alternative bifurcation (green), the remaining alternatives (red), and conflict or support that have <50% bootstrap support (gray). Number above and below branches represent the number of concordant and discordant informative gene trees, respectively.
