# Supplementary material for: Phylogenomic analyses in Phrymaceae reveal extensive gene tree discordance in relationships among major clades
Source: Am J Bot. 2022 Jun 5;109(6):1035–46. doi: 10.1002/ajb2.1860 (PMC9328367; doi:10.1002/ajb2.1860)
Supplement: Supplementary file 6 — Appendix S6. Species network inferred from PhyloNet maximum likelihood analyses with one to three maximum reticulations of the reduced data sets. (A) Phrymaceae backbone. (B) Erythranthe cardinalis, E. lewisii, and E. bicolor. Red and blue branches indicate the minor and major edges, respectively, of hybrid nodes. Numbers next to colored branches indicate inheritance probabilities for each hybrid node. [file AJB2-109-1035-s010.pdf]

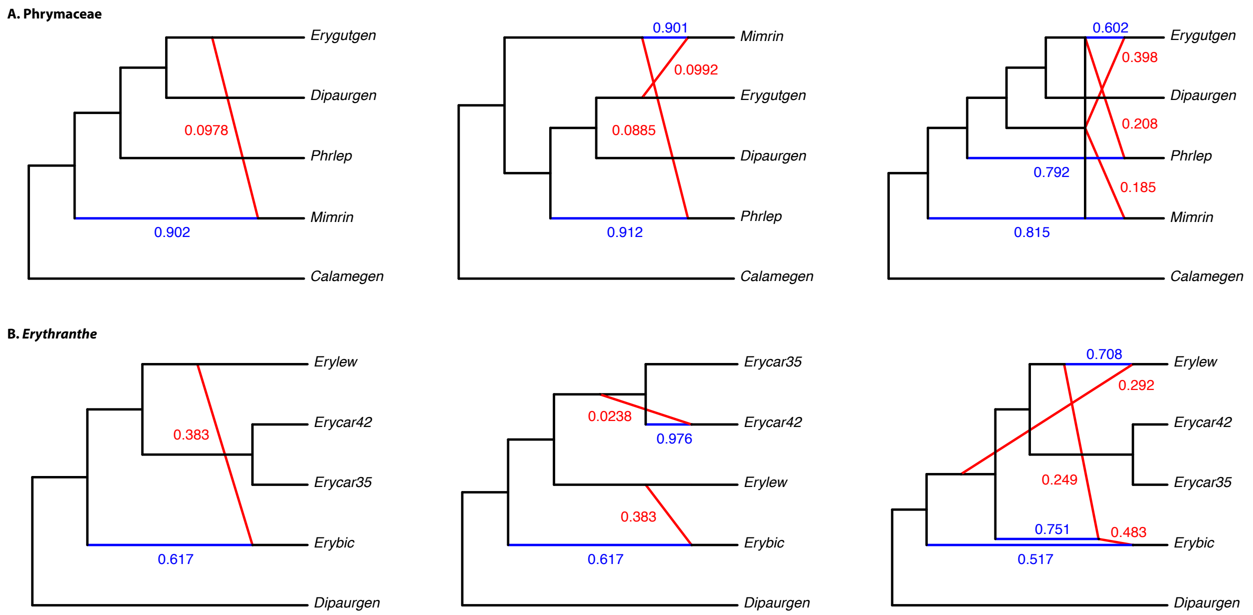

Appendix S6. Species network inferred from PhyloNet maximum likelihood analyses with one to three maximum reticulations of the reduced data sets. A) Phrymaceae backbone. B) *Erythranthe cardinalis*, *E. lewisii*, and *E. bicolor.* Red and blue branches indicate the minor and major edges, respectively, of hybrid nodes. Numbers next to colored branches indicate inheritance probabilities for each hybrid node.
