# Supplementary material for: Phylogenomic analyses in Phrymaceae reveal extensive gene tree discordance in relationships among major clades
Source: Am J Bot. 2022 Jun 5;109(6):1035–46. doi: 10.1002/ajb2.1860 (PMC9328367; doi:10.1002/ajb2.1860)
Supplement: Supplementary file 9 — Appendix S9. 95% credibility set for the reduced taxon set of Erythranthe cardinalis, E. lewisii, and E. bicolor from Bayesian inference in PhyloNet. (A) The maximum posterior probability (MPP) network representing 54% of the credibility set. (B) The second most frequent network (30%). (C) The third most frequent network (13.5%). Red and blue branches indicate the minor and major edges, respectively, of hybrid nodes. Numbers next to colored branches indicate inheritance probabilities for each hybrid node. [file AJB2-109-1035-s007.pdf]

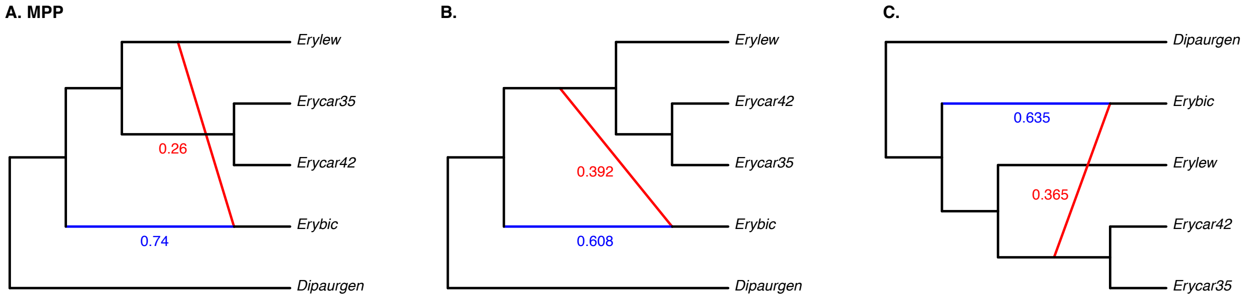

Appendix S9. 95% credibility set for the reduced taxon set of *Erythranthe cardinalis*, *E. lewisii*, and *E. bicolor* from Bayesian inference in Phylonet. A. The maximum posterior probability (MPP) network representing 54% of the credibility set. B. Second most frequent network (30%). C. Third most frequent network (13.5%). Red and blue branches indicate the minor and major edges, respectively, of hybrid nodes. Numbers next to colored branches indicate inheritance probabilities for each hybrid node.
