# Supplementary material for: Phylogenomic analyses in Phrymaceae reveal extensive gene tree discordance in relationships among major clades
Source: Am J Bot. 2022 Jun 5;109(6):1035–46. doi: 10.1002/ajb2.1860 (PMC9328367; doi:10.1002/ajb2.1860)
Supplement: Supplementary file 10 — Appendix S10. Maximum likelihood cladogram of Phrymaceae inferred with IQ‐TREE from the concatenated 732‐nuclear gene supermatrix. Numbers above branches are gene duplication counts and numbers below branches are gene duplication percentages. Numbers next to species names are haploid chromosome numbers. All chromosome counts are from the Chromosome Counts Database (Rice et al., 2015), except Erythranthe pardalis (Nesom, 2012). When multiple independent counts gave a single consistent chromosome number but different counts were each reported by a single study, we ignored the outlier numbers. Inset: Histogram of percentages of gene duplication per branch. [file AJB2-109-1035-s009.pdf]

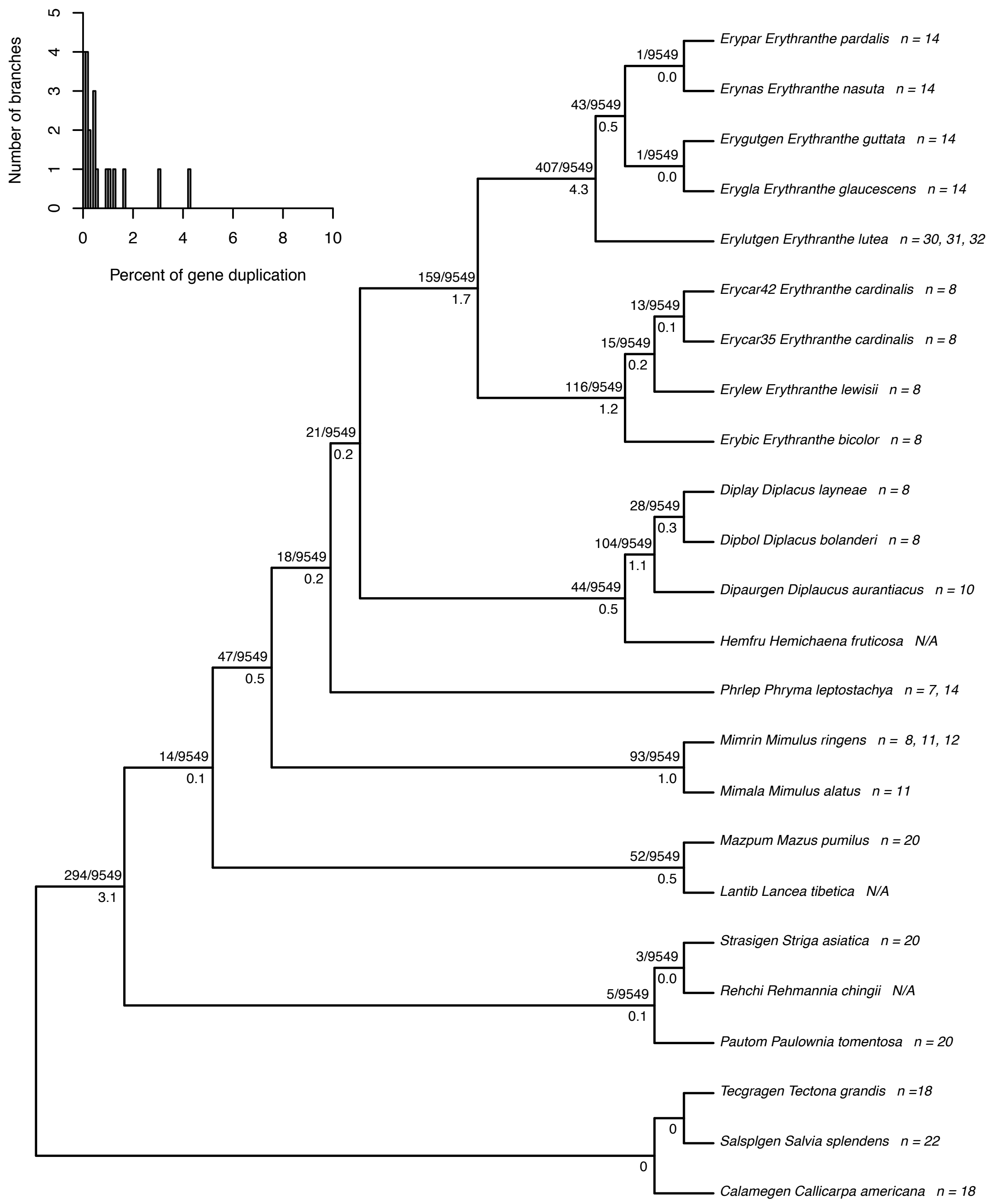

Appendix S10. Maximum likelihood cladogram of Phrymaceae inferred with IQ-TREE from the concatenated 732-nuclear gene supermatrix. Numbers above branches are gene duplication counts and numbers below branches are gene duplication percentages. Numbers next to species names are gametophytic chromosome counts. All chromosome counts are from the Chromosome Counts Database (Rice et al., 2015), except *Erythranthe pardalis* (Nesom, 2012). When multiple independent counts gave a single consistent chromosome number but different counts were each reported by a single study, we ignored the outlier numbers. Inset: Histogram of percentages of gene duplication per branch.
