# Supplementary material for: Phylogenomic analyses in Phrymaceae reveal extensive gene tree discordance in relationships among major clades
Source: Am J Bot. 2022 Jun 5;109(6):1035–46. doi: 10.1002/ajb2.1860 (PMC9328367; doi:10.1002/ajb2.1860)
Supplement: Supplementary file 11 — Appendix S11. Distribution of synonymous distance among gene pairs (Ks) for each genome or transcriptome. (A) Distribution of raw Ks values between 0 and 3. (B) Distribution of Ks values zooming in to between 0 and 0.5. (C) Plots of log‐transformed Ks values. Colored lines indicate components inferred using a mixture model. Blue lines indicate a component from an ancestral whole genome triplication event early in core eudicots; red lines are from more recent whole genome or small‐scale duplication events. [file AJB2-109-1035-s006.pdf]

a)

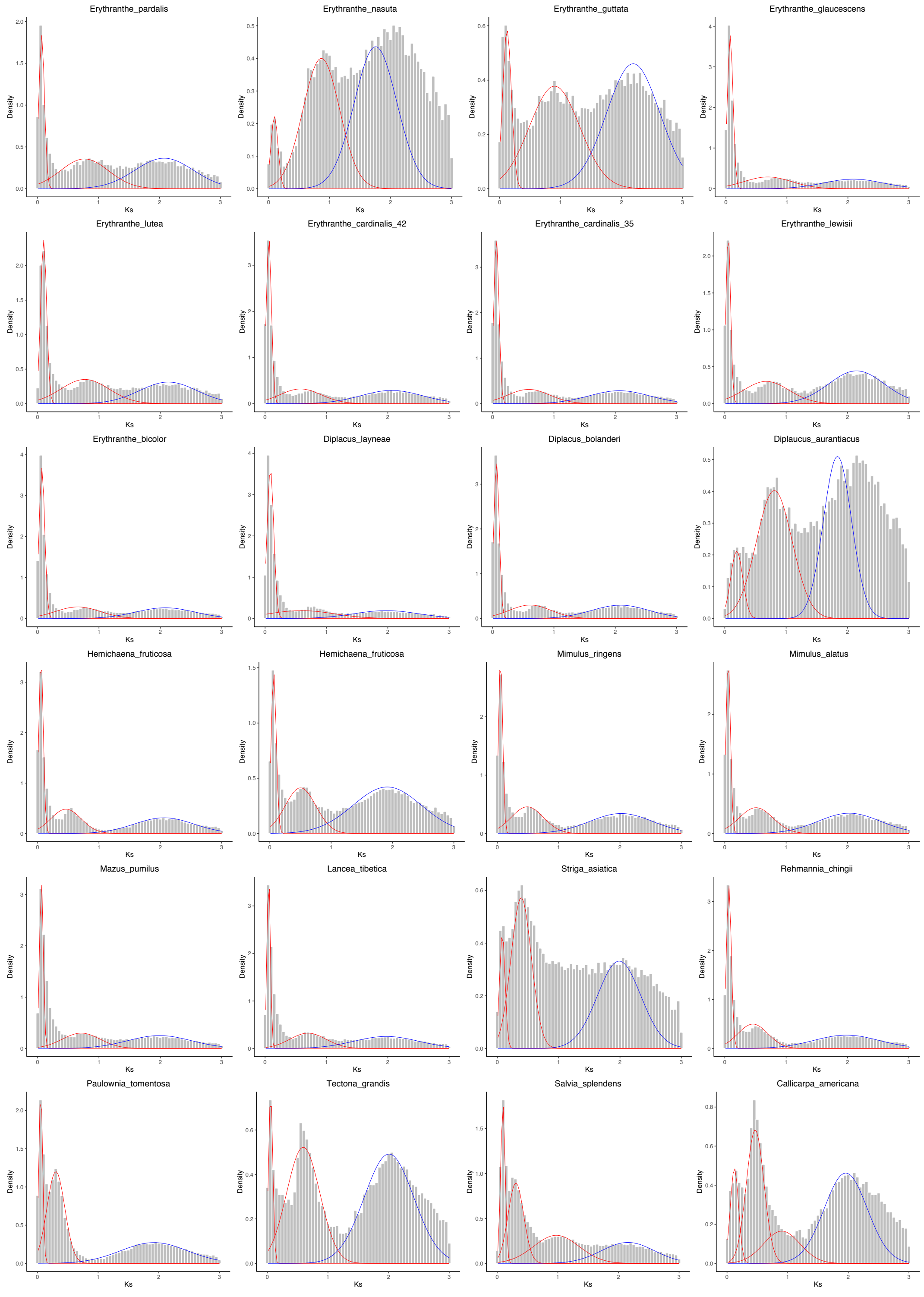
Erythranthe_pardalis
Erythranthe_nasuta
Erythranthe_guttata
Erythranthe_glaucescens
Erythranthe_lutea
Erythranthe_cardinalis_42
Erythranthe_cardinalis_35
Erythranthe_lewisii
Erythranthe_bicolor
Diplacus_layneae
Diplacus_bolanderi
Diplaucus_aurantiacus
Hemichaena_fruticosa
Hemichaena_fruticosa
Mimulus_ringens
Mimulus_alatus
Mazus_pumilus
Lancea_tibetica
Striga_asiatica
Rehmannia_chingii
Paulownia_tomentosa
Tectona_grandis
Salvia_splendens
Callicarpa_americana
Density
Ks

b)

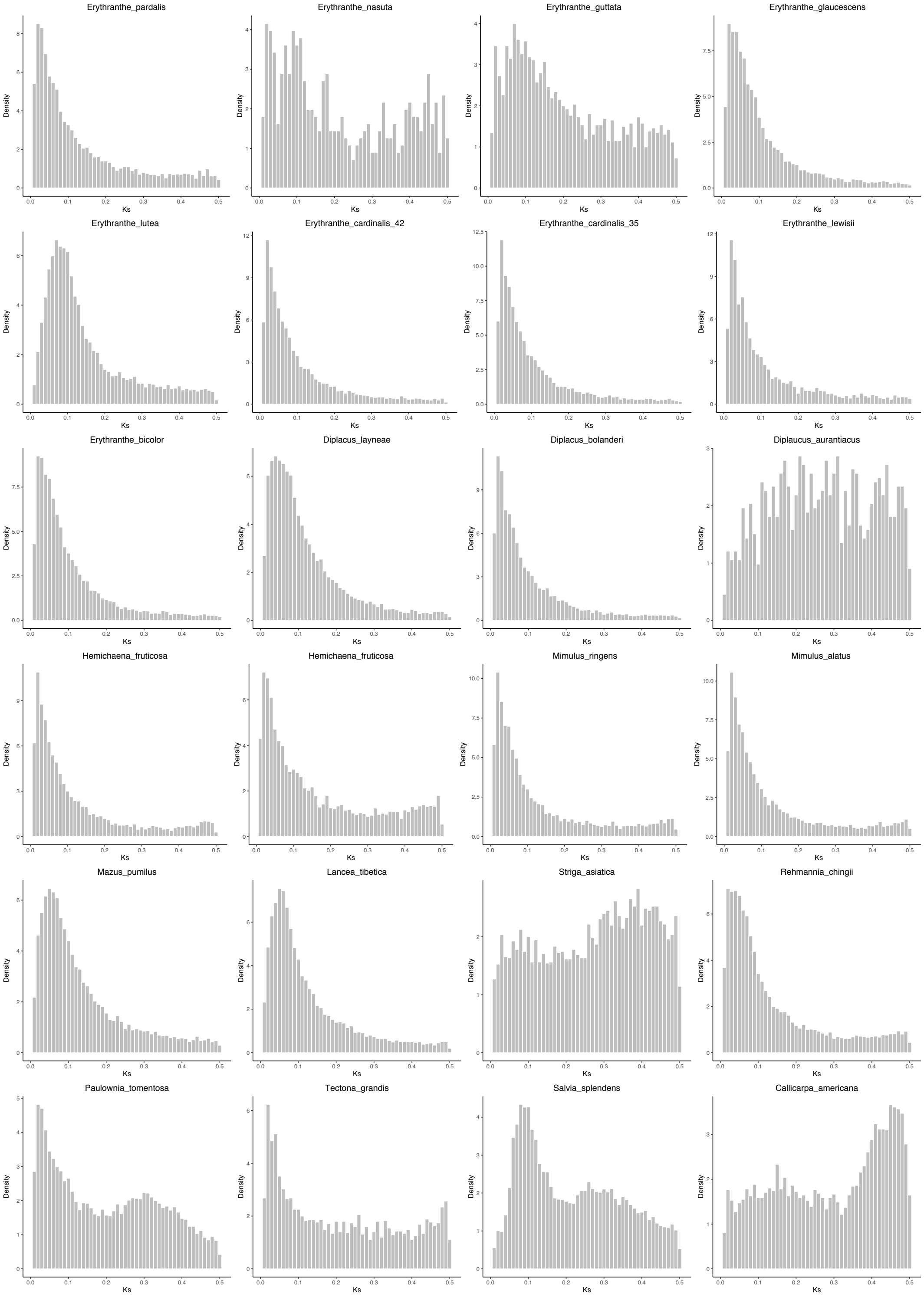
Erythranthe_pardalis
Erythranthe_nasuta
Erythranthe_guttata
Erythranthe_glaucescens
Erythranthe_lutea
Erythranthe_cardinalis_42
Erythranthe_cardinalis_35
Erythranthe_lewisii
Erythranthe_bicolor
Diplacus_layneae
Diplacus_bolanderi
Diplaucus_aurantiacus
Hemichaena_fruticosa
Hemichaena_fruticosa
Mimulus_ringens
Mimulus_alatus
Mazus_pumilus
Lancea_tibetica
Striga_asiatica
Rehmannia_chingii
Paulownia_tomentosa
Tectona_grandis
Salvia_splendens
Callicarpa_americana
Density
Ks

c)

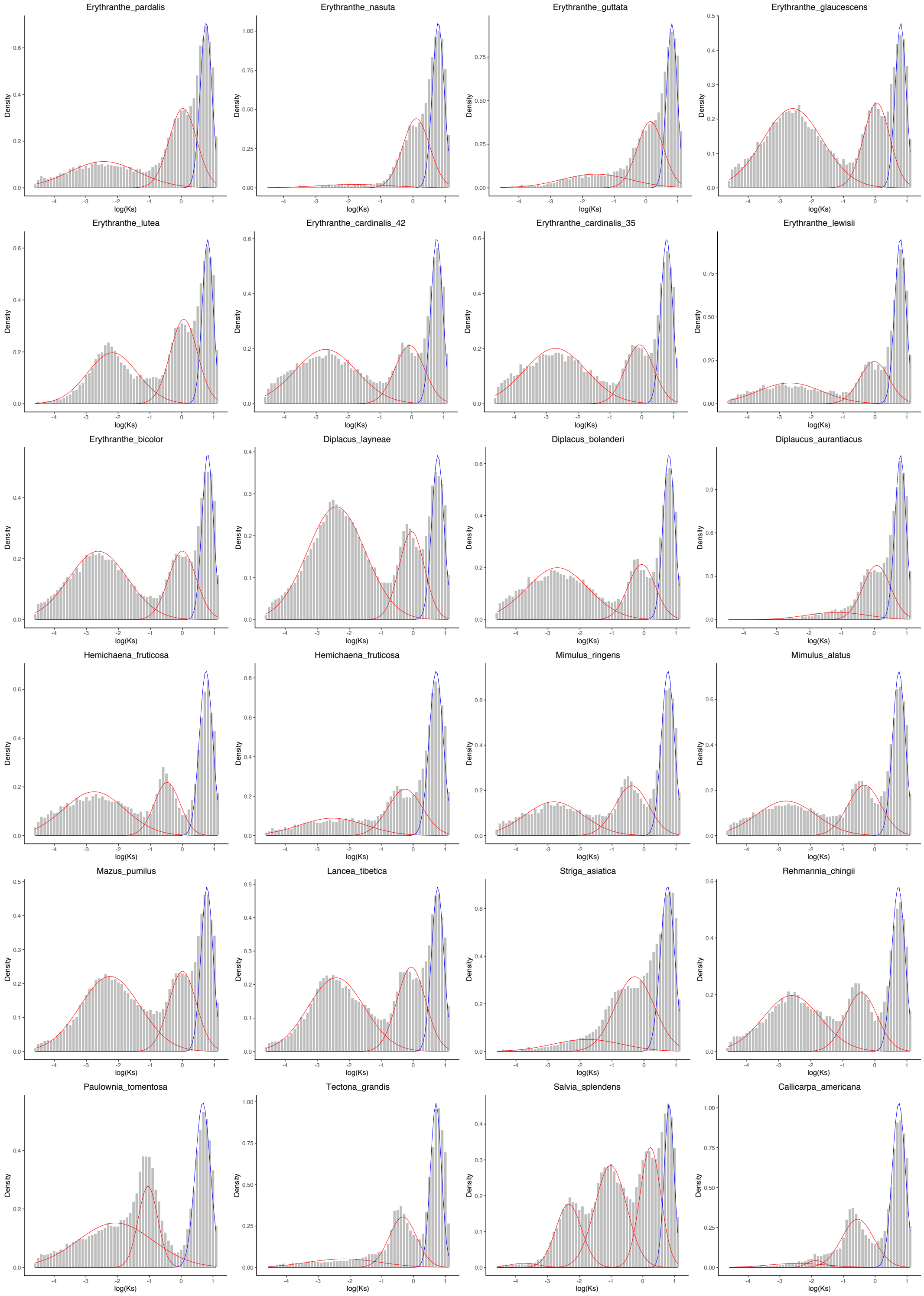
Erythranthe_pardalis
Erythranthe_nasuta
Erythranthe_guttata
Erythranthe_glaucescens
Erythranthe_lutea
Erythranthe_cardinalis_42
Erythranthe_cardinalis_35
Erythranthe_lewisii
Erythranthe_bicolor
Diplacus_layneae
Diplacus_bolanderi
Diplaucus_aurantiacus
Hemichaena_fruticosa
Hemichaena_fruticosa
Mimulus_ringens
Mimulus_alatus
Mazus_pumilus
Lancea_tibetica
Striga_asiatica
Rehmannia_chingii
Paulownia_tomentosa
Tectona_grandis
Salvia_splendens
Callicarpa_americana
Density
log(Ks)

Appendix S11. Distribution of synonymous distance among gene pairs (Ks plots) for each genome or transcriptome. A) Plots of raw Ks distances between 0 and 3. B) Plots of Ks distances zooming in to values between 0 and 0.5. C) Plots of log-transformed Ks distances. Colored lines indicate components inferred using a mixture model. Blue lines indicate a component from an ancestral whole genome triplication event early in core eudicots; red lines are from more recent whole genome or small-scale duplication events.
